# Comparison of chemical compounds and their influence on the taste of coffee depending on green beans storage conditions

**DOI:** 10.1038/s41598-022-06676-9

**Published:** 2022-02-17

**Authors:** Magdalena Zarebska, Natalia Stanek, Krzysztof Barabosz, Anna Jaszkiewicz, Renata Kulesza, Rafał Matejuk, Dariusz Andrzejewski, Łukasz Biłos, Artur Porada

**Affiliations:** 1 The Łukasiewicz Research Network-Institute of Heavy Organic Synthesis “Blachownia”, Energetykow 9, 47-225 Kedzierzyn-Kozle, Poland; 2 Przedsiebiorstwo Handlowo Usługowe P.A.R.K. Katarzyna Porada, Plac Teatralny 12, 45-056 Opole, Poland; 3 Faculty of Mechanical Engineering, Opole University of Technology, Proszkowska 76, 45-758 Opole, Poland; 4 Faculty of Production Engineering and Logistics, Opole University of Technology, Proszkowska 76, 45-758 Opole, Poland

**Keywords:** Chemistry, Engineering

## Abstract

Currently, there is no technology for the storage of green coffee (GrC), that results in obtaining high-quality roasted coffee (RC). The aim of this study was to evaluate the effects of storage temperature (− 10, 5, 10, 18, 20 °C), postharvest treatment method (natural (N), washed (W)) and type of packaging material (GrainPro (G), jute (J) bags) on the content of chlorogenic acids (CQAs), caffeine and trigonelline as well as the sensory profile of RC from the specialty sector after 12 months of regulated storage. Sensory analysis showed that natural coffees have better taste and higher quality than washed coffees after 12 months of storage. The highest total scores were obtained from the natural coffee stored in a GrainPro bag at − 10 °C followed by coffee stored in a jute bag at 10 °C which had the smallest decreases compared to the initial recorded values. No notable differences among CQA contents in washed coffees stored in either type of bag was seen but natural coffees stored in jute bags at 10 °C and 18 °C displayed the lowest drops relative to the initial values.

## Introduction

Coffee is one of the most widely consumed products worldwide. In the 2018/2019 season, global coffee production reached 170.22 million (60-kilo) bags which was 4.6% higher than that in the 2017/2018 season. The production of Arabica was approximately 100, while Robusta accounted for 70 million bags^1^. Since improving cup quality and changing the image of coffee by propagating information about its health benefits, coffee consumption has increased significantly. People are increasingly conscious about what they consume and want to know where their coffee comes from, is its origin, and how it is roasted^2^. Roasteries have noticed this trend and have therefore brought out their special coffee products.

Generally, not all beans can be used for high-quality coffee. According to the Specialty Coffee Association of America (SCAA), coffee with a total score of 80 points or more in the sensory analysis is classified as “specialty”^3^. Judges assess important flavour attributes of coffee according to the Cupping Specialty Coffee Protocol: aroma, flavour, aftertaste, acidity, body, balance, uniformity, clean cup, sweetness, and defects. The specialty coffee market is quite young compared to the entire global coffee market. The specialty coffee market is distinguished by its approach to the raw material, which consists of both the coffee plantation and green and roasted beans. Throughout every stage of production, from cultivation to the cup of brewed coffee, all conditions are controlled. Coffee trees grow in only rural areas, and the fruits are harvested only by hand so that only ripe cherries are picked. Then, the cherries are processed carefully under controlled conditions by various methods, e.g., natural (dry, when the entire cherry is first cleaned and then dried) and washed (wet, when the fruits covering the beans are removed before drying). Afterwards, the beans are dried to the appropriate humidity level and then transported to their destination, i.e., a coffee roastery where the beans are roasted in a craft way to bring out their full aroma and flavour. The recipients of such beans are specialty cafes that have perfected the entire coffee preparation process. Specialty coffees are distinguished not only by their full aroma and unique flavour but also by their chemical composition^4,5^. Sanmiguel, Torrez & Pérez-Villarreal indicated that over the last decade, consumer interest in the behaviour and sensory attributes of coffee has increased significantly^6^. Additionally, Barahona, Sanmiguel & Yang showed the relationship between the sensory attributes of coffee beverages and the price of coffee^7^.

A few years ago, consumers mainly perceived coffee as a stimulant; today, they are informed about the beneficial components and suggested health benefits. Regular coffee drinkers are less likely to suffer from a range of chronic and degenerative diseases (e.g., cancer, cardiovascular disorders, diabetes, and Parkinson’s) than people who do not drink coffee^8–10^. The positive health effects of coffee consumption are attributed to the presence of caffeine, trigonelline, and chlorogenic acids^11^. Caffeine is the most widely consumed psychoactive substance worldwide and has many biological effects such as its ability to stimulate and maintain cognitive function^12^. Trigonelline is the second most predominant alkaloid in raw coffee beans. The roasting of coffee beans converts trigonelline to nicotinic acid, a water-soluble B vitamin also known as niacin. Trigonelline appears to have several biological activities including acting as an antimicrobial agent and regenerating dendrites and axons in cortical neurons^13^. Moreover, trigonelline may indirectly affect hypoglycaemia and hypolipidaemia^14^. Various studies have also indicated that coffee has high concentrations of polyphenols^15–17^. The most studied phenolic compounds in coffee are chlorogenic acids. Among these isomers, 3-*O*-caffeoylquinic acid is thought to be the most abundant in coffee^18,19^. Chlorogenic acids are considered to have positive effects on the prevention of diabetes and the treatment of its symptoms^20^.

Harvesting processes, roast degree, and GrC bean storage conditions are responsible for the production or degradation of several compounds, which give the coffee beverages their sensory characteristics^19^. Keeping coffee fresh is critical to preserving taste. During inappropriate GrC bean storage, there is a decrease in the quality and levels of the chemical components, which is expressed as a flattening of the cup quality. Abreu, Boréma, Oliveirab, Almeidac & Alvesa indicated that changes in the chemical composition of green coffee beans occur in the different packaging materials during storage. Beans stored in paper packages that are permeable to gases and water vapour (similar to jute bags) experience greater decreases in quality. According to the authors, after 12 months, beans stored in this manner could no longer be considered specialty coffee beans^21^. The results of Tripetch & Borompichaichartkul also showed that an HDPE bag can better preserve the amount of chlorogenic acid in green coffee beans during storage than a jute sack^22^. Coradi, Borem, Saath, & Marques^23^ noticed that washed coffee presents better quality than the product in its natural form. High temperatures and a high water content have been reported as the main factors that cause sensory changes in coffee during storage^5,24,25^.

GrC beans spend a lot of time in storage before they reach the consumer. The literature indicates^22,26,27^ that the storage conditions of raw beans are an important parameter that influences the quality of coffee beverages, but there is a lack of studies about the optimal conditions for GrC storage and their influence on the sensory characteristics and chemical composition of the coffee. Ensuring that the storage areas have the optimal conditions helps preserve the rich taste, safety, and quality of GrC beans. Due to the long storage time and high transport costs of coffees from the specialty sector, preservation of the chemical compounds present in GrC beans seems to be crucial for specialty coffee roasters and for consumers who have gotten used to the taste of coffee. That is why an increasing number of coffees are contracted for 12 and even up to 24 months because coffee from the same plantation may taste different in the next harvest. Finding the best storage conditions may not only slow down the degradation process of the bean but also be the driving force behind the construction of a central, fully controlled warehouse for more than one coffee roaster. Central storage would also allow for consolidated purchases of raw materials, which can be beneficial for both farmers and roasters. Therefore, in this study, we evaluated the influence of GrC bean storage conditions on the chemical composition and cup quality. GrC beans were stored for 12 months in measured chambers at various temperatures (− 10, 5, 10, 18, and 20 °C) and in different packaging materials (polypropylene-GrainPro and natural fibre-jute bags) to determine their quality attributes, including taste loss during storage and the levels of caffeine, trigonelline and chlorogenic acids. The need to find the best temperature and type of packaging for GrC bean storage is therefore warranted to allow the good properties of the green beans and roasted grain to be maintained.

## Results and discussion

### Acronyms

**Bean type: GrC**-green coffee, **RC**-roasted coffee;

**Bag type: G**-GrainPro, **J**-jute;

**Post-harvest treatment: N**-natural, **W**-washed;

**Coffee sample labels: GW**-GrainPro washed, **GN**-GrainPro natural, **JW**-jute washed, **JN**-jute natural;

**Compounds: 5-CQA**-5-O-caffeoylquinic acid, **3-CQA**-3-O-caffeoylquinic acid, **4-CQA**-4-O-caffeoylquinic acid.

#### Effects of the storage temperature and type of packaging on the sensory quality of coffee beans

One of the most important factors influencing consumer approval of a product is its sensory features. The cupping form provides important flavour attributes for coffee: aroma, flavour, aftertaste, acidity, body, balance, uniformity, clean cup, sweetness, and overall. All of these attributes are rated on a 16-point scale that represents the level of quality in quarter-point increments with numeric values ranging from 6 to 10, where 6.00–6.75 is good, 7.00–7.75 is very good, 8.00–8.75 is excellent and 9.00–9.75 is outstanding. The final score is calculated by summing the individual scores given for each attribute and additionally for a clean cup, uniformity, and sweetness. The final score allows coffee to qualify as specialty coffee when it is above 80 points^3^.

**Figure 1:**
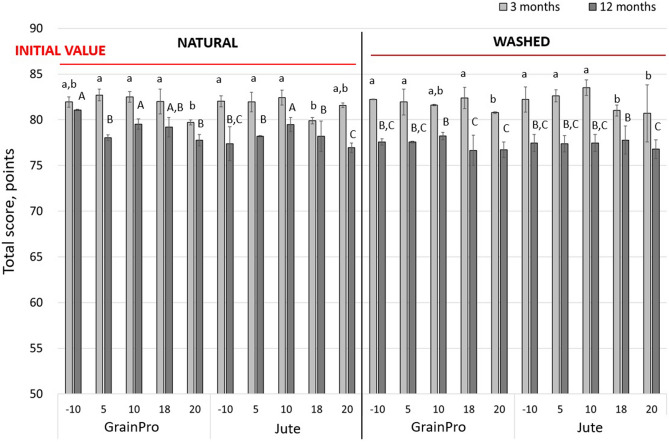
Comparison of the total scores for the coffee samples roasted from washed and natural processed green beans stored in GrainPro and jute bags for 3 and 12 months relative to the initial values before storage. The superscripts a and b denote significant (*p* < 0.05) differences between the coffees stored in different bags and at different temperatures for 3 months. The superscripts A, B, C denote significant (*p* < 0.05) differences between the coffees stored in different bags and at different temperatures for 12 months. Mean values ± standard deviation (n = 10).

After receiving the coffee in Poland, the first sensory evaluation, the so-called calibration, took place the day before the coffee samples were put into temperature chambers. At this time, all of the attributes for the natural coffee brew El Oregano (86.0 points total score) and washed coffee brew Finca La Maravilla (86.8 points total score) were determined (Fig. 1, red line). The results confirmed the specialty quality of both coffees and were confirmed by Coradi, Borem, Saath, & Marques^23^, who noticed that washed coffee presents better quality than the product in its natural form.

**Figure 2:**
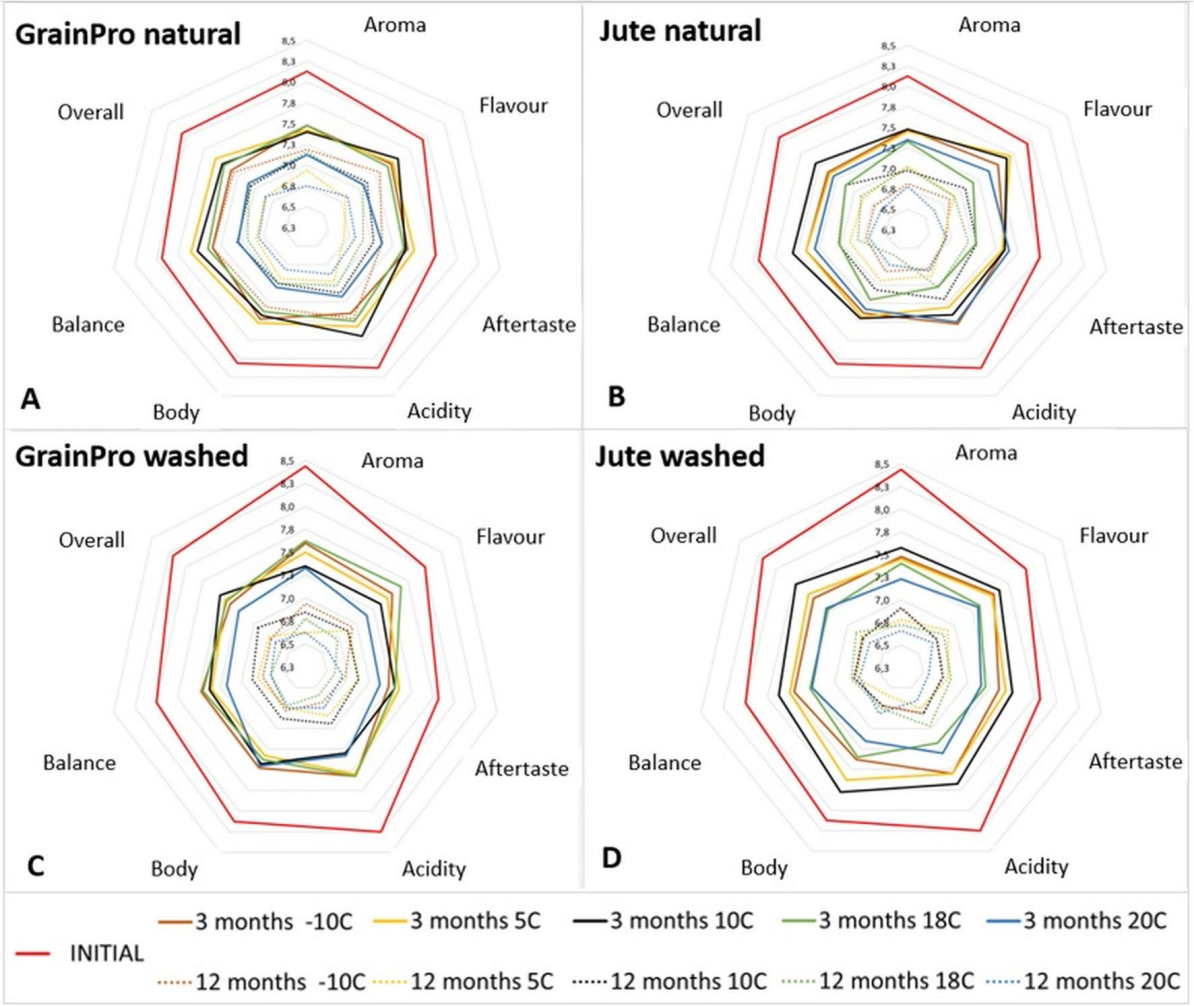
Sensory profiles of the roasted coffee samples: natural (**A**, **B**) and washed (**C**, **D**) processing and stored in GrainPro (**A**, **C**) and jute (**B**, **D**) bags after 3 and 6 months of storage compared to the initial values before storage. Mean values (n = 10).

The results of the sensory evaluation regarding the taste characteristics are graphically presented as radar charts (Fig. 2). Figure 2 illustrates the changes that took place in RC from the beginning of sensory evaluation (initial value, red line) to 3 and finally 12 months of storage, taking into account the type of bag and method of coffee bean processing.

At the beginning of the sensory evaluation for the natural processed coffees in the chambers, all attributes remained at similar levels (7.8–8.1 points), with the aroma and acidity being the most noticeable and the aftertaste being the least noticeable (Fig. 2, red line). For the washed coffees, there were similar distinguishable attributes, but with greater differentiation (7.8–8.4 points).

The next sensory evaluation of the coffees stored in the chambers was performed 3 months after both types of coffees were stored at the indicated temperatures. Then, every three months for one year, all of the sensorial attributes were determined. The results at 0, 3, and 12 months of storage are further described. After 3 months of storage at the tested temperatures, the highest total score (Fig. 1, light grey bars) was observed for the washed processed coffee stored in a jute bag in a 10 °C chamber (83.5 points). This value was 3.3 points lower than the corresponding initial value. However, a higher, 5–7% decrease from the initial value was observed for washed processed coffees in general. For natural coffees, these decreases were smaller and accounted for 4–5% of the initial value. High total score values were noted for natural coffees stored in the GrainPro bags from the 5 and 10 °C chambers (82.7 and 82.5 points) as well as for that stored in the jute bags at 10 °C (82.4 points).

The coffees stored for 3 months maintained their high quality with scores greater than 80 points. The exceptions were the natural coffees from the jute 18 °C and GrainPro 20 °C chambers, for which the lowest total score values were recorded (79.9 and 79.7 points, respectively); these values were significantly (*p* < 0.05) different from the other coffees stored for 3 months and lower than their initial value by more than 6 points. Considering the sensory attributes individually, we found that the attributes that displayed the greatest decreases after 3 months of storage in relation to the initial values were aroma and acidity (Fig. 2, continuous lines). In natural coffees, these decreases were smaller (5–10%), while in washed coffees, they were 8–14% lower. The losses in flavour, aftertaste, and balance in both types of processed coffee were similar and did not exceed 8%, except for the chambers at temperatures of 18 and 20 °C, where the decreases from the initial values were 14%. In general, the coffees stored at lower temperatures (− 10, 5, and 10 °C) retained a higher taste quality than the coffees stored at 18 and 20 °C. In the higher temperature chambers, the losses were 7% that of the initial value in terms of the total score and 14% those of aroma and acidity. A lower quality decline was noticed for natural processed coffees. Results found in this study were observed by Pérez-Martínez, Sopelana, Peña & Cid^28^, who noticed slower and less pronounced changes in the refrigeration temperature. Their coffee infusions lost their aroma intensity and freshness, while also acquiring some undesirable notes, such as a rancid aroma, mainly during storage at 25 °C. Rojas^29^ reported a similar conclusion that coffee with a moisture content above 15% retains its quality at temperatures as low as 10 °C.

After 12 months in the chambers, the natural and washed coffees began to differentiate. Natural processed coffees showed higher quality properties in terms of individual sensory characteristics (Fig. 2, dotted lines) and hence total scores (Fig. 1, dark grey bars), which were slightly below 80 points. The highest total score was achieved for the naturally processed coffee samples stored in GrainPro bags in − 10 °C and 10 °C chambers (81.1 and 79.5 points) and in jute bags at 10 °C (79.5 points). These declines are merely 6–8% that of the initial total scores. Significantly lower (*p* < 0.05) scores were recorded for the washed processed coffees stored in chambers 18 °C and 20 °C in both, jute and GrainPro bags. These decreases amounted to approximately 10 points relative to the initial total score values, which equates to a loss of approximately 12%. Among the analysed attributes, aroma and acidity were the features that suffered the greatest losses in washed coffees. This confirms the trend of 3-month storage. However, this time, the losses were greater and amounted to 21% that of the initial values, especially in the 18 and 20 °C chambers. Changes of up to 19% in body and overall attributes were also observed. For both postharvest coffee treatments, the smallest decreases (up to 14%) in sensory values compared to the initial values were noted for the aftertaste, flavour, and balance. After 12 months of storage, the acidity and overall attributes play the main roles in the assessment of taste. The 10 °C chamber provided the highest taste values among the individual sensory attributes after 12 months of storage between the considered types of bags and postharvest treatments. Especially for the natural coffees stored in jute bags, the taste values after 12 months were comparable to those obtained after 3 months of storage (except for the aroma). Only natural coffee stored in a GrainPro bag at − 10 °C had higher values.

#### Effects of storage temperature and type of packaging on the contents of chlorogenic acids, caffeine, and trigonelline

**Figure 3:**
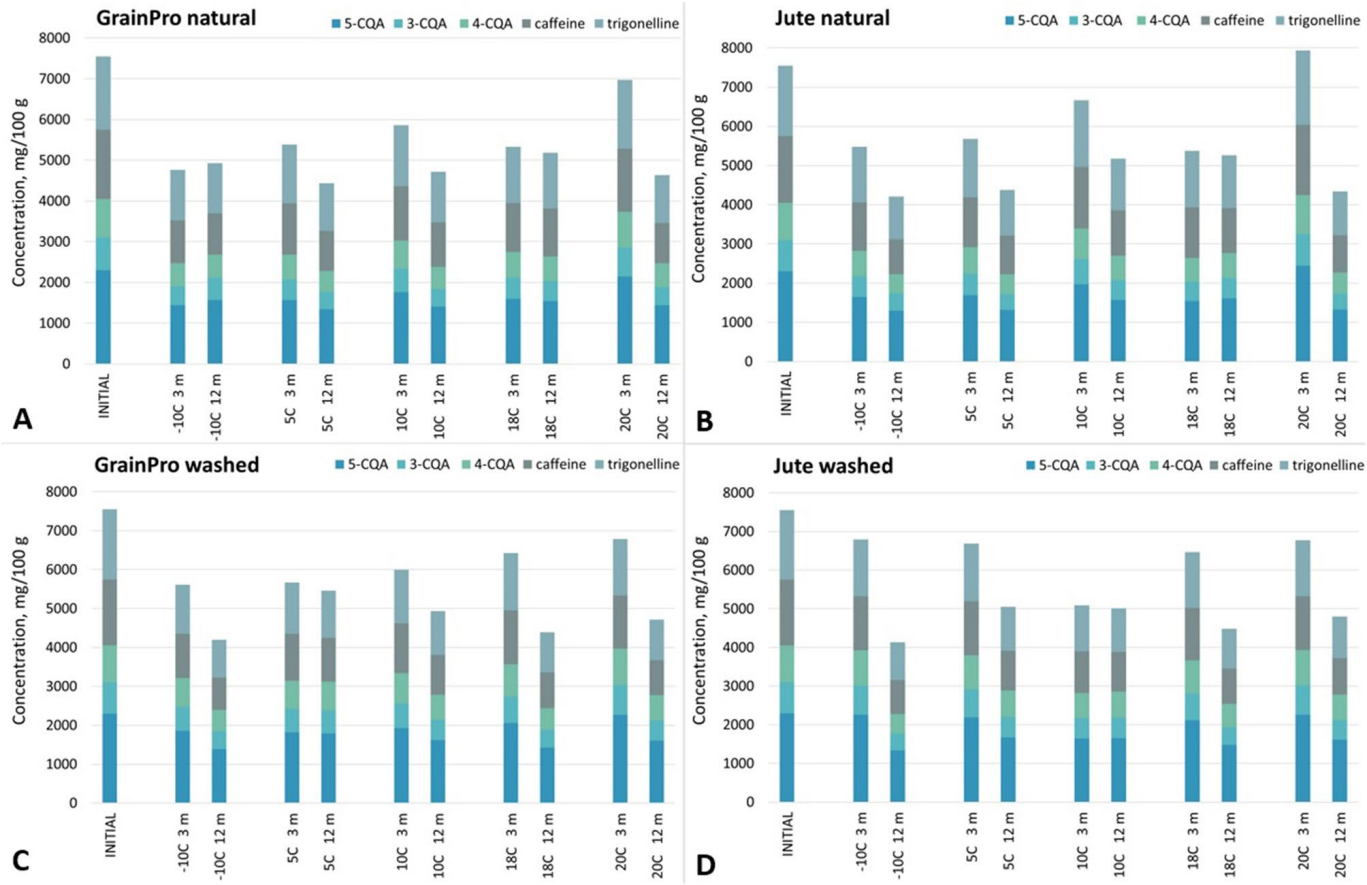
Comparison of compound concentrations (in mg/100 g) found in the coffee samples roasted from washed (**C**, **D**) and natural (**A**, **B**) processed green beans stored in GrainPro (**A**, **C**) and jute (**B**, **D**) bags for 3 and 12 months relative to the initial values of the coffees before storage. 5-CQA (5-O-caffeoylquinic acid), 4-CQA (4-O-caffeoylquinic acid), 3-CQA (3-O-caffeoylquinic acid). Mean values (n = 3).

The aim of this part of the study was to separate, identify, and quantify the important coffee extract substances, divided into two groups of chemical compounds. The first group consisted of polyphenolic acids, namely, chlorogenic acids such as 3-O-caffeoylquinic acid (3-CQA), 4-O-caffeoylquinic acid (4-CQA), and 5-O-caffeoylquinic acid (5-CQA), while the second group consisted of alkaloids, of which caffeine and trigonelline are the key components. After delivery to Poland, the green coffee beans were first analysed by HPLC the day before the coffee samples were placed in the temperature chambers. The next HPLC analysis was performed after 3 months of storage and the last 12 months of storage at the indicated temperatures. The results at 0, 3, and 12 months of storage are presented in Fig. 3 and Table 1.

**Table 1:** The levels of identified chemical compounds (mg/100 g) in the initial roasted coffee and after storage for 3 and 12 months.

Time (months)	Bag/processing	Temperature [°C]	Concentration [mg/100 g]				
			5-CQA	3-CQA	4-CQA	Caffeine	Trigonelline
0	N		2301 ± 72	803 ± 12	948 ± 17	1709 ± 28	1796 ± 41
	W		2499 ± 50	856 ± 16	1056 ± 20	1599 ± 32	1611 ± 26
3	GN	− 10	1440 ± 85	465 ± 11	565 ± 15	1052 ± 76	1244 ± 72
		5	1568 ± 110	500 ± 16	611 ± 26	1267 ± 50	1440 ± 48
		10	1761 ± 67	573 ± 7	698 ± 10	1326 ± 26	1504 ± 18
		18	1598 ± 28	519 ± 3	632 ± 7	1202 ± 11	1377 ± 8
		20	2142 ± 38	715 ± 6	874 ± 7	1551 ± 14	1688 ± 20
	JN	− 10	1646 ± 29	530 ± 4	648 ± 7	1236 ± 16	1417 ± 21
		5	1695 ± 81	552 ± 4	668 ± 7	1273 ± 14	1486 ± 9
		10	1968 ± 133	643 ± 10	784 ± 16	1568 ± 30	1703 ± 25
		18	1544 ± 36	493 ± 2	605 ± 7	1288 ± 13	1449 ± 15
		20	2442 ± 99	811 ± 31	995 ± 34	1789 ± 86	1899 ± 85
	GW	− 10	1852 ± 125	615 ± 10	745 ± 17	1142 ± 25	1254 ± 27
		5	1823 ± 78	602 ± 9	726 ± 12	1201 ± 27	1321 ± 11
		10	1927 ± 46	636 ± 5	776 ± 9	1280 ± 19	1374 ± 9
		18	2058 ± 38	678 ± 4	831 ± 11	1388 ± 3	1467 ± 7
		20	2269 ± 63	767 ± 8	936 ± 13	1364 ± 15	1455 ± 7
	JW	− 10	2256 ± 84	754 ± 7	916 ± 12	1391 ± 8	1475 ± 2
		5	2191 ± 65	724 ± 5	880 ± 8	1403 ± 29	1489 ± 12
		10	1640 ± 46	532 ± 3	646 ± 4	1077 ± 25	1197 ± 7
		18	2111 ± 50	700 ± 5	853 ± 8	1354 ± 16	1444 ± 12
		20	2263 ± 150	754 ± 10	916 ± 19	1384 ± 31	1459 ± 23
12	GN	− 10	1567 ± 25	553 ± 16	562 ± 11	1010 ± 17	1232 ± 29
		5	1343 ± 7	426 ± 5	515 ± 8	980 ± 11	1170 ± 8
		10	1405 ± 47	440 ± 22	540 ± 22	1093 ± 35	1243 ± 60
		18	1544 ± 26	489 ± 11	607 ± 11	1178 ± 19	1361 ± 20
		20	1437 ± 17	447 ± 8	584 ± 28	990 ± 18	1181 ± 17
	JN	− 10	1294 ± 88	448 ± 56	487 ± 38	890 ± 67	1092 ± 72
		5	1314 ± 12	410 ± 6	505 ± 8	985 ± 12	1170 ± 9
		10	1574 ± 29	500 ± 16	624 ± 13	1157 ± 19	1325 ± 25
		18	1612 ± 11	516 ± 8	638 ± 6	1149 ± 10	1342 ± 9
		20	1324 ± 71	407 ± 25	540 ± 39	948 ± 55	1121 ± 48
	GW	− 10	1388 ± 58	458 ± 22	543 ± 26	836 ± 38	973 ± 34
		5	1790 ± 110	594 ± 37	734 ± 52	1123 ± 75	1215 ± 86
		10	1614 ± 28	526 ± 13	644 ± 13	1025 ± 18	1121 ± 14
		18	1427 ± 8	459 ± 6	556 ± 3	920 ± 7	1029 ± 3
		20	1607 ± 22	516 ± 8	650 ± 12	900 ± 14	1041 ± 8
	JW	− 10	1335 ± 19	429 ± 9	518 ± 8	871 ± 12	979 ± 10
		5	1669 ± 71	543 ± 34	673 ± 29	1033 ± 40	1133 ± 36
		10	1649 ± 7	540 ± 9	665 ± 5	1037 ± 12	1121 ± 7
		18	1481 ± 18	464 ± 5	591 ± 13	919 ± 13	1033 ± 2
		20	1610 ± 36	516 ± 13	654 ± 19	943 ± 25	1080 ± 20

**GN**—GrainPro natural, **JN**—jute natural, **GW**—GrainPro washed, **JW**—jute washed, **5-CQA**—5-O-caffeoylquinic acid, **4-CQA**—4-O-caffeoylquinic acid, **3-CQA**—3-O-caffeoylquinic acid. Mean value ± standard deviation (n = 3).

#### Chlorogenic acids

The washed processed coffees contained significantly (*p* < 0.05) higher contents of chlorogenic acids (approximately 4400 mg/100 g) than the natural processed coffees (approximately 4000 mg/100 g) before storage. Additionally, 5-CQA was the dominant form among the chlorogenic acids. Both of these observations are consistent with previous from Leloup, Cancel, Liardon, Rytz, & Pithon^30^ and Farah, & Donangelo^31^ for coffee in general. After three months of storage, the highest content of chlorogenic acids was recorded in the natural coffee stored in a jute bag at 20 °C (approximately 4250 mg/100 g). However, this result was an exception, as the other natural processed coffees represented significantly (*p* < 0.05) lower values of CQAs (2470–3730 mg/100 g) in general than the washed coffees (2818–3972 mg/100 g). Additionally, storage in jute bags was more favourable for washed processed coffees except for stored at 10 °C. The decreases in the contents of chlorogenic acids compared with the initial values were also the smallest in jute bags (18%). For the washed coffees stored in GrainPro bags, a 28% loss in CQAs was recorded. Apart from the 18 °C chamber and JW, a certain temperature trend was seen. The higher the storage temperature was, the more chlorogenic acids were retained in the coffee beans. In general, after 12 months of storage, higher levels of chlorogenic acids were found in the washed coffees. The exceptions were the − 10 °C chambers, where low values for both types of bags were recorded and the largest declines from the initial values were noticed (up to 48%). Additionally, natural processed coffees from GrainPro bags stored at − 10 °C and from jute bags stored at 10 and 18 °C presented high values. The losses compared with the initial values for these coffee samples were the lowest at approximately 30%. No visible temperature trend was seen after 12 months of storage. Significant differences (*p* < 0.05) between the washed and natural postharvest treatment methods during 12 months of storage were observed. Higher CQA contents were noted in wet-processed Arabica seeds compared with those in dry-processed seeds, which could have been caused by the loss of other water-soluble components from leaching and wet fermentation^32^. However, the degradation of these ingredients in the dry processed seeds exposed to sunlight was much lower. This tendency was visible throughout the 12 months of storage. The higher chemical stability of chlorogenic acids in plants stored at higher compared to lower temperatures may be due to the selective induction and repression of enzymes by temperature, time, substrate availability or a combination of these factors in phenol biosynthesis. The action of inducers and repressors, such as heat, carbohydrates or enzymes, may regulate phenol biosynthetic activity^37^. The decrease in CQA contents during storage could have occurred due to enzymatic and nonenzymatic oxidation^22^ and the participation of these compounds in other simultaneous oxidation reactions, such as lipid oxidation, since phenolic compounds are well-known radical scavengers^33^. In addition, the decrease in the concentration of 5-CQA suggests that it acted as an antioxidant, to minimize protein and lipid oxidation^34^. The results showed that the CQA content of natural and washed processed coffees differed from the beginning of storage until 12 months.

#### Caffeine

In natural processed coffees at the beginning of storage, the caffeine content was approximately 1700 mg/100 g, while in their washed counterparts, it was approximately 1600 mg/100 g. After the washed processed coffees were stored for 3 months, the caffeine contents ranged from 1077 to 1384 mg/100 g, while in natural processed coffee, the caffeine contents were in the range of 1052–1789 mg/100 g of coffee. Smaller decreases compared with the initial values were observed for washed processed coffees, especially those stored in jute bags. The greatest loss of caffeine was recorded in the natural processed coffees, particularly those stored in GrainPro bags. The mean decrease in caffeine content compared with the initial value for both treatments was approximately 20%. After 12 months of storage, the lowest caffeine content was recorded for the washed processed coffees stored in − 10 °C chamber in both types of bags (approximately 850 mg/100 g). The highest value was observed for the natural processed coffees stored at 10 and 18 °C (approximately 1150 mg/100 g). The caffeine contents in the naturally processed samples were in the range of 890–1178 mg/100 g while the washed coffees were between 836 and 1123 mg/100 g of coffee.

After 12 months of storage, for natural and washed processed coffees approximately 40% decrease in relation to the initial value in caffeine content was noted. Additionally, the storage temperature of green beans had no effect on the caffeine content of the stored beans.. This is in agreement with other authors^30,35^ and can be explained by the thermo stability of caffeine. Additionally, no temperature trends were found for the tested storage time.

#### Trigonelline

The trigonelline level in the natural processed coffees before storage was 1796 mg/100 g, while in their washed counterparts, this value was 1611 mg/100 g. After three months of storage, the highest content of trigonelline was recorded in the natural coffee stored in a jute bag at 20 °C (approximately 2000 mg/100 g). However, in general, the smallest losses occurred in washed coffees, and jute bags better preserved this alkaloid (approximately 9% loss in relation to the initial value). The highest trigonelline contents were noticed for the natural processed coffees stored for 12 months in both types of bags at 10 and 18 °C (1243–1342 mg/100 g). These conditions help to preserve more trigonelline in the coffee grains (only an approximately 25% decline). Comparing these values to those for the − 10 °C chamber containing washed processed coffees stored in both types of bags, an approximately 40% decrease relative to the initial value was observed.

For the tested storage time, no visible correlation between storage temperature and trigonelline content was found, which indicates the stability of this alkaloid. Additionally, no significant differences between the tested seed processing methods were noted.

### Statistical analysis

#### Effects of storage temperature and type of packaging on the correlation between chemical and sensory variables of coffee beans

A correlation matrix was used to find significant correlations between the considered variables (Table 2). This compilation allowed us to observe that an increase in the storage temperature caused a loss of flavour and aroma of the coffee brews while increasing the 4-CQA content. In addition, jute bags affected the body attribute. The washed postharvest process showed a relationship with the content of CQAs, while the natural process showed a relationship with the other parameters; furthermore, the trigonelline content was higher. All CQAs were strongly positively correlated with each other and more weakly correlated with caffeine, whereas 4-CQA further contributed to the aroma. The caffeine content strongly corresponded to trigonelline and affected the aftertaste, acidity, and overall attributes, while the trigonelline content was strongly correlated with cup quality, which is in agreement with the literature^35^.

**Table 2:** Statistical correlations between individual parameters.

	Temperature [°C] (− 10, 5, 10, 18, 20)	Bag (G/J)	Grain preparation (N/W)	5-CQA*	3-CQA*	4-CQA*	Caffeine	Trigonelline
5-CQA*	0.294	− 0.134	− 0.413	1.000	0.948	0.952	0.461	0.266
	*p* = 0.163	*p* = 0.532	*p* = 0.045		*p* = 0.000	*p* = 0.000	*p* = 0.023	*p* = 0.209
3-CQA*	0.029	− 0.177	− 0.404	0.948	1.000	0.832	0.346	0.186
	*p* = 0.895	*p* = 0.409	*p* = 0.050	*p* = 0.000		*p* = 0.000	*p* = 0.098	*p* = 0.384
4-CQA*	0.471	− 0.069	− 0.411	0.952	0.832	1.000	0.479	0.269
	*p* = 0.020	*p* = 0.749	*p* = 0.046	*p* = 0.000	*p* = 0.000		*p* = 0.018	*p* = 0.203
Caffeine	0.352	− 0.004	0.458	0.461	0.346	0.479	1.000	0.932
	*p* = 0.092	*p* = 0.985	*p* = 0.024	*p* = 0.023	*p* = 0.098	*p* = 0.018		*p* = 0.000
Trigonelline	0.300	− 0.057	0.712	0.266	0.186	0.269	0.932	1.000
	*p* = 0.155	*p* = 0.792	*p* = 0.000	*p* = 0.209	*p* = 0.384	*p* = 0.203	*p* = 0.000	
Aroma	− 0.423	− 0.1680	0.550	− 0.246	− 0.150	− 0.414	0.265	0.405
	*p* = 0.039	*p* = 0.433	*p* = 0.005	*p* = 0.247	*p* = 0.486	*p* = 0.044	*p* = 0.211	*p* = 0.050
Flavour	− 0.423	− 0.354	0.426	0.083	0.208	− 0.107	0.342	0.455
	*p* = 0.040	*p* = 0.090	*p* = 0.038	*p* = 0.700	*p* = 0.329	*p* = 0.620	*p* = 0.102	*p* = 0.026
Aftertaste	− 0.190	− 0.345	− 0.396	0.296	0.356	0.152	0.523	0.571
	*p* = 0.373	*p* = 0.099	*p* = 0.055	*p* = 0.161	*p* = 0.088	*p* = 0.479	*p* = 0.009	*p* = 0.004
Acidity	− 0.2358	− 0.261	0.548	0.094	0.177	− 0.077	0.415	0.549
	*p* = 0.267	*p* = 0.218	*p* = 0.006	*p* = 0.664	*p* = 0.408	*p* = 0.719	*p* = 0.043	*p* = 0.005
Body	− 0.3175	− 0.455	0.484	− 0.088	0.033	− 0.246	0.155	0.338
	*p* = 0.131	*p* = 0.026	*p* = 0.016	*p* = 0.683	*p* = 0.879	*p* = 0.246	*p* = 0.469	*p* = 0.106
Balance	− 0.369	− 0.345	0.468	0.068	0.184	− 0.118	0.323	0.448
	*p* = 0.076	*p* = 0.099	*p* = 0.021	*p* = 0.753	*p* = 0.390	*p* = 0.585	*p* = 0.124	*p* = 0.028
Overall	− 0.300	− 0.307	0.475	0.137	0.219	− 0.035	0.432	0.535
	*p* = 0.154	*p* = 0.144	*p* = 0.019	*p* = 0.522	*p* = 0.303	*p* = 0.872	*p* = 0.035	*p* = 0.007

Bold results: correlation coefficient with an absolute value greater than 0.4 and a *p* value < 0.05. **G**—GrainPro bag, **J**—jute bag, **N**—natural, and **W**—washed processed coffee bean, **5-CQA**—5-O-caffeoylquinic acid, **4**-**CQA**—4-O-caffeoylquinic acid, **3-CQA**—3-O-caffeoylquinic acid.

#### Effects of storage temperature and type of packaging on the differentiation between roasted coffee brews in terms of sensory and chemical discriminants

**Figure 4:**
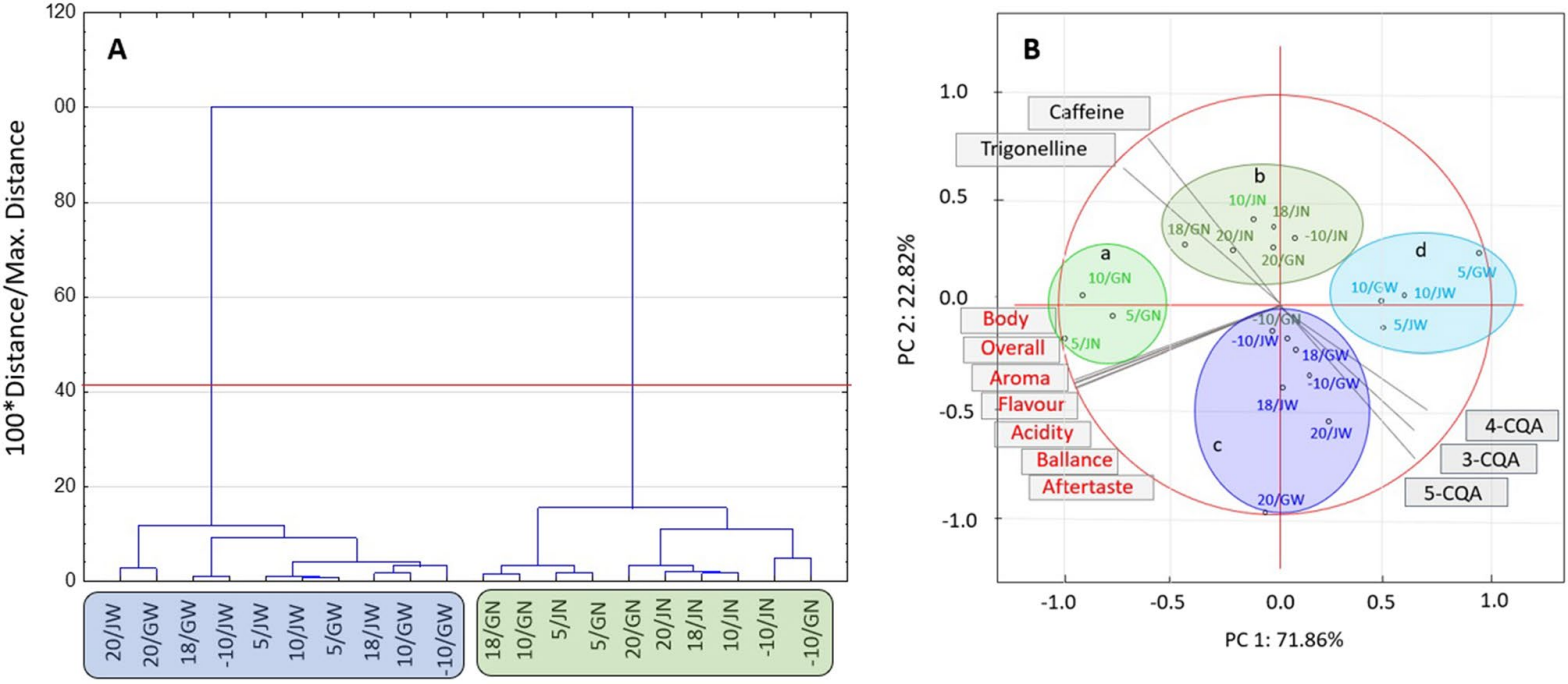
Chemical and sensory discriminants for the roasted coffees stored for 12 months. (**A**) Tree diagram. (**B**) Biplot of the projection of variables into the factor plane (Statistica, ver. 10, StatSoft). GW-GrainPro washed, GN-GrainPro natural, JW-jute washed, JN-jute natural, RC-roasted coffee.

Analysis of the factor loading matrix of variables in the factor space showed that the first component consists of all highly negatively correlated (> 0.94) sensory attributes, although this correlation was slightly weaker for trigonelline and 4-CQA was positively correlated. The second principal component contained caffeine (positive) and 5-CQA as well as 3-CQA (negative). Figure 4 represents the cluster analysis (CA) and principal component analysis (PCA) of the chemical and sensorial characteristics of roasted coffee grains after 12 months of storage under different conditions. The presented dendrogram (Fig. 4A) graphically shows the partitioning of the analysed roasted coffees in terms of their postharvest treatment method into two groups: the natural processing group (green) and the washed processing group (blue).

A similar tendency was found in the PCA (Fig. 4B), taking into consideration the data obtained for CQAs, caffeine, trigonelline, and sensory characteristics. PCA allowed us to visually divide the analysed coffee samples into two sectors: natural with negative PC1 and positive PC2 values and washed with positive PC1 and negative PC2 values. The factorial arrangement of the coffee sample objects allowed for the identification of 4 groups: a. roasted coffees derived from a natural process and stored in GrainPro or jute bags in 5 and 10 °C chambers (light green dots); b. roasted coffees derived from a natural process and stored in both types of bags in − 10, 18 and 20 °C chambers (dark green dots); c. roasted coffees derived from the washed processing method stored in GrainPro and jute bags in − 10, 18, and 20 °C chambers (dark blue dots); and d. roasted coffees derived from the washed processing method stored in both types of bags in 5 and 10 °C chamber (light blue dots). Two main factors with eigenvalues greater than one explained more than 94.7% of the data variance.

The first distinguishing factor (PC1) explained nearly 71.9% of the data variance, and its eigenvalue of 8.6 indicated that it contains information originally explained by nearly 9 variables used to describe the research object (7 sensory attributes, trigonelline, and 4-CQA). The second distinguishing factor (PC2) explained nearly 22.8% of the data variance and translates the information of the other three variables (3-CQA, 5-CQA, and caffeine). The location of the roasted coffee extract group from JN and GN at 5 and 10 °C (ellipse a) indicates greater importance of the discriminants related to the high content of trigonelline and all sensory attributes, which translates into a high total score. In contrast to the light blue group (ellipse d), where the presence of 4-CQA is more important, the caffeine and 5-CQA contents within both groups fluctuated at similar levels. In the case of both washed and natural RCs from ellipses b and c, the determined components had values with a similar level of differentiation along with the first principal component. In the b group, infusions with high caffeine importance and lower 5-CQA contents can be distinguished as opposite to the c group.

In this work, it was shown that grain postharvest treatment, packaging material, and temperature affected the sensory attributes, chlorogenic acids, caffeine, and trigonelline contents during 12 months of storage.

Fresh, unstored, washed processed coffees induced greater taste sensations than natural coffees. After 3 months of storage, the Arabica coffee from Guatemala still maintained high quality, as illustrated by the total sensory score of 80 points. After 12 months of storage, the washed coffee lost its quality, and the naturally processed coffees were characterized by a better taste and higher quality properties in terms of the individual sensory characteristics; hence, the total scores for these coffees were slightly below 80 points. The highest score in terms of general quality was awarded to the natural coffee stored in the − 10 °C chamber in the GrainPro bag. However, large fluctuations in sensory attributes were recorded for the coffees stored in other types of packaging in this chamber, and the lower profitability of maintaining such a low temperature in large coffee warehouses is unfavourable. Comparably high quality and taste benefits were presented by the natural coffee stored in a 10 °C chamber in jute bags. In this case, the decrease in taste values compared to the initial values before storage was 8%, the most noticeable tastes were acidity and overall, and the total score in the qualitative classification was 79.5 points. The lowest results were observed at 20 °C with a 12% drop. A score > 79 can be defined to be almost at a specialty level, but certainly, these are premium quality coffees, which constitute a relatively low percentage of the total coffee production worldwide.

Statistical analysis showed that natural coffees stored in both types of bags at 5 and 10 °C and their washed counterparts differed mainly in terms of their different sensory attributes and contents of 5-CQA and trigonelline. On the other hand, the diversity among the natural and washed coffees stored in both types of bags at − 10, 18, and 20 °C was mainly influenced by the contents of 4-CQA, 3-CQAs.

Washed processed coffees contain significantly more chlorogenic acids than natural caffees after roasting both before and after 3 months of storage. After this time, the sum of CQAs in the washed coffees stored in jute bags was higher than that in GrainPro bags. At the end of storage time, there was no visible difference between the CQA levels in the washed coffees stored in jute or GrainPro bags. However the jute bags seems to be more favourable for storing natural processed coffee at 10 and 18 °C because these samples presented the lowest drops relative to the initial value. At the end of storage, drops of approximately 40% for caffeine and 30% for trigonelline in relation to the initial value were observed. After 12 months of storage, no significant differences in the levels of both alkaloids from the natural and washed processed coffees stored in different types of bags and at different temperatures were noted.

In particular, the type of packaging material should be taken into account during the long-term storage of green coffee beans. A commonly used, traditional and inexpensive from green grain storage that is easily adaptable for the small scale, and reusable is the jute bag. An alternative form of packaging is GrainPro bags, a polypropylene equivalent that exhibits good barrier properties.

## Methods

### Coffee samples

Arabica (*Coffea arabica* L.) green coffee beans were delivered from Finca El Oregano (1700 masl)-natural processed coffee and Finca La Maravilla (1850 masl)-washed processed coffee plantations located in Huehuetenango, Guatemala. Both types of coffee were dried until reaching 11% (wb) moisture and sealed in GrainPro or jute bags. 5 Months after harvesting, the coffees arrived in Poland by sea.

A statement on guidelines as experimental research and field studies on plants (either cultivated or wild), is comply with relevant institutional, national, and international guidelines and legislation. Studies comply with local and national regulations—formal ethical approval is not required.

#### Reagents

Analytical standards of chlorogenic acid (5-CQA, CAS: 327-97-9, 5-O-caffeoylquinic acid), neochlorogenic acid (3-CQA, CAS: 906-33-2, 3-O-caffeoylquinic acid), caffeine (CAS: 58-08-2), and trigonelline (CAS: 535-83-1) were purchased from Sigma-Aldrich, Fluka, ChromaDex. All standards used were of analytical grade (≥ 99% purity). The 5-CQA standard was used to quantify both 5-CQA and 4-CQA. Mobile phase was prepared by diluting formic acid (CAS: 64-18-6, Fisher Scientific, LC–MS purity) with ultrapure water (Direct-Q system, resistivity below 18 MΩ cm) to a concentration of 0.1% (w/w) and the pH was adjusted to 2.4. The solution was filtered under a vacuum system through a 0.45 µm filter. The second part of the mobile phase was methanol (CAS: 67-56-1, POCH, HPLC grade). Stock solutions of 1000 mg/l were prepared by diluting each analysed compound in HPLC grade methanol. These stock solutions were diluted in methanol to reach intermediate concentrations. All solutions were stored in the refrigerator at 4 °C.

#### Coffee storage

After arrival in Poland, the green coffee beans were divided into 100 g samples and packed separately into small GrainPro or jute bags. In this form, they were stored in measured chambers at various temperatures (− 10, 5, 10, 18, and 20 °C) for 12 months. The humidity level was maintained at 50% relative humidity (RH) except for the − 10 °C chamber, which had a humidity of 39% RH. After delivering the coffee to Poland, the first sensory evaluation (so-called calibration) and HPLC measurements took place the day before the coffee samples were put into the temperature chambers. The next sensory evaluation and HPLC survey of the coffees stored in the chambers were performed 3 months after placing both types of coffees at the indicated temperatures. Then, every three months for 12 months, all of the sensorial attributes and HPLC-measured chemical components were determined. This study presents the results at 0, 3, and 12 months.

#### Roasting

After 12 months of storage, green coffee bean samples were removed from each measured chamber for stabilization. Half were used for further chemical analysis, while the other half were roasted after a day of stabilization. A ROEST S100 sample roaster designed for optimal workflow and efficiency in a coffee lab maintained the following conditions: roasting start temperature: 165 °C, final temperature: 205 °C, roasting time: 6–7 min with a fixed airflow and heater setting, and development time: 53 s. The heat application during roasting was the same for each sample in each stage of the roasting process. Moreover, the time from the first crack until the end of the roasting (development) was the same (53 s). The colour check was performed using a Probat Colorette 4 measuring device. Then, the roasted coffee beans were subjected to sensory and chemical analyses.

#### Extraction

For each experiment, approximately 120 g of green and roasted coffee beans were ground (Blender type GB25, Hamilton Beach Commercial, USA). From such a homogenized coffee sample, three independent extractions were completed using 0.5 g of milled beans and 15 mL of distilled water (90 °C) with the assistance of shaker (Multi-Tube Vortexer, Benchmark, USA) for 10 min (500 rpm). Furthermore, the sample solution was filtered using 0.45 µm nylon with a glass prefilter (GF/NY, PureLand) before analysis. Each sample extraction was performed in triplicate.

#### HPLC analysis

Chromatographic analysis was performed on a Dionex UltiMate 3000 chromatograph equipped with an automatic pump, injector, autosampler, column compartment, and UV–VIS detector with photodiode array (PDA) technology. Instrument control, data acquisition and processing were managed with Chromeleon 6.8 software. Chlorogenic acids (3-CQA, 4-CQA, 5-CQA), trigonelline and caffeine were quantitatively determined according to the method described in our previous work^36^.

#### Sensory analysis

Sensory evaluation of the coffee samples was performed using the scaling method described by the SCAA (2015). Five certified judges in two independent cuppings evaluated seven sensorial attributes (aroma, flavour, aftertaste, acidity, body, balance, and overall impression). The green coffee beans were roasted 48 h before cupping according to the roasting conditions described above. Then, the samples were ground immediately before cupping and no more than 15 min before water infusion. Hot water (97 °C) was poured directly into the measured grounds to the rim of the cup. The steeping time occurred 4 min before the evaluation process started.

#### Statistical analysis

All HPLC results in the study are expressed as the mean from three independent replicates measured in duplicate (n = 3) ± the standard deviation. For sensory analysis, the results from 5 cuppers in 2 independent cuppings using triangulation were used and these results are also expressed as the mean (n = 10) ± standard deviation. Mean values were compared by ANOVA and a Tukey HDS post hoc test. Classification techniques, such as cluster analysis (CA; tree diagram using Ward’s method with Euclidean distance) and principal component analysis (PCA), were used to interpret the obtained results. Calculations were performed using the software Statistica (www.statsoft.pl) ver. 10. A correlation matrix was used to find a significant correlation between the considered variables. Differences were considered significant when the p value was < 0.05.
